# Identification of Predictive DNA Methylation Biomarkers for Chemotherapy Response in Colorectal Cancer

**DOI:** 10.3389/fphar.2017.00047

**Published:** 2017-02-13

**Authors:** Rashidah Baharudin, Nurul-Syakima Ab Mutalib, Sri N. Othman, Ismail Sagap, Isa M. Rose, Norfilza Mohd Mokhtar, Rahman Jamal

**Affiliations:** ^1^UKM Medical Molecular Biology Institute, Universiti Kebangsaan MalaysiaKuala Lumpur, Malaysia; ^2^Department of Surgery, Faculty of Medicine, Universiti Kebangsaan MalaysiaKuala Lumpur, Malaysia; ^3^Department of Clinical Oral Biology, Faculty of Dentistry, Universiti Kebangsaan MalaysiaKuala Lumpur, Malaysia; ^4^Department of Physiology, Faculty of Medicine, Universiti Kebangsaan MalaysiaKuala Lumpur, Malaysia

**Keywords:** epigenetics, DNA methylation, colorectal cancer, 5-fluorouracil, 5-aza-2′-deoxycytidine, chemoresistance, recurrent cancer

## Abstract

Resistance to 5-Fluorouracil (5-FU) is a major obstacle to the successful treatment of colorectal cancer (CRC) and posed an increased risk of recurrence. DNA methylation has been suggested as one of the underlying mechanisms for recurrent disease and its contribution to the development of drug resistance remains to be clarified. This study aimed to determine the methylation phenotype in CRC for identification of predictive markers for chemotherapy response. We performed DNA methylation profiling on 43 non-recurrent and five recurrent CRC patients using the Illumina Infinium HumanMethylation450 Beadchip assay. In addition, CRC cells with different genetic backgrounds, response to 5-FU and global methylation levels (HT29 and SW48) were treated with 5-FU and DNA methylation inhibitor 5-aza-2′-deoxycytidine (5-azadC). The singular and combined effects of these two drug classes on cell viability and global methylation profiles were investigated. Our genome-wide methylation study on the clinical specimens showed that recurrent CRCs exhibited higher methylation levels compared to non-recurrent CRCs. We identified 4787 significantly differentially methylated genes (*P* < 0.05); 3112 genes were hyper- while 1675 genes were hypomethylated in the recurrent group compared to the non-recurrent. Fifty eight and 47 of the significantly hypermethylated and hypomethylated genes have an absolute recurrent/non-recurrent methylation difference of ≥20%. Most of the hypermethylated genes were involved in the MAPK signaling pathway which is a key regulator for apoptosis while the hypomethylated genes were involved in the PI3K-AKT signaling pathway and proliferation process. We also demonstrate that 5-azadC treatment enhanced response to 5-FU which resulted in significant growth inhibition compared to 5-FU alone in hypermethylated cell lines SW48. In conclusion, we found the evidence of five potentially biologically important genes in recurrent CRCs that could possibly serve as a new potential therapeutic targets for patients with chemoresistance. We postulate that aberrant methylation of *CCNEI, CCNDBP1, PON3, DDX43*, and *CHL1* in CRC might be associated with the recurrence of CRC and 5-azadC-mediated restoration of 5-FU sensitivity is mediated at least in part by MAPK signaling pathway.

## Introduction

Colorectal cancer (CRC) is the third most common cancer in worldwide contributing to 10% of the total new cancer cases and 9% of cancer deaths in 2012 (Ferlay et al., 2015). The high incidence and mortality demonstrate the global CRC burden. The disease burden is similar in Malaysia where CRC is the second most common cancer diagnosed among males and third common cancer in females. According to the National Cancer Registry (NCR) Report in the year 2011, 2246 CRC cases were reported in 2007, contributing to 12.3% of total cancer cases (Zainal Ariffin and Nor Saleha, 2011). Of these, 1185 cases were males and 1101 cases females.

To date, various treatment strategies have been used to treat CRC depending on the stage at which cancer was discovered. In the early stages of CRC (Stages I and II), surgery is the best option (Holen and Chung, 2008). However, for advanced stages such as stage III and stage IV, treatment often consist of a combination of therapies such as surgery, chemotherapy and/or radiotherapy to minimize the risk of recurrence (Hayat, 2009). Managing CRC remains a clinical obstacle, as more than 15% of cases are resistant to 5-Fluorouracil (5-FU)-based chemotherapeutic regimens which is the mainstay of treatment, and tumor recurrence rates can be as high as 50–60% (Shakibaei et al., 2014). Furthermore, most of recurrence cases occur in the first 3 years after a surgery and the majority started to recur in the second year (Aghili et al., 2010). Various factors have been proposed to play a significant role in CRC recurrence and one of the factors is chemotherapy resistance (Kanwar et al., 2012).

Epigenetic alterations have been suggested as one of the underlying mechanisms of chemotherapy resistance (Zeller and Brown, 2010). One major epigenetic mechanism involved in the progression of cancer is DNA methylation which refer to the enzymatic addition of a methyl group to the 5′ position of cytosine ring by DNA methyltransferase (DNMT) to produce 5-methylcytosine (Bestor, 2000). Altered DNA methylation patterns can influence the expression of the genes by silencing or activating the genes. In general, there are two types of aberrant DNA methylation that could contribute to the development and progression of cancer which are global DNA hypomethylation and CpG island hypermethylation (Wajed et al., 2001).

The term DNA hypomethylation refers to global decrease in the level of methylation in the genome of tumor cells in comparison to normal tissue (Lao and Grady, 2011). On the contrary, hypermethylation is an increase of DNA methylation level at CpG island as compared to normal tissue. Methylation of CpG islands within the promoter of CRC results in silencing of tumor suppressor genes and promote tumor formation (Herman and Baylin, 2003). Methylation of CpG islands at particular genes may also contribute to the acquisition of drug resistance. This was demonstrated by Arnold et al. (2003) whereby methylation of CpG island in the MLH1 gene is associated with resistance to 5-FU and development of chemoresistance could increase the incidence of recurrence CRC. However, this process is reversible and could be achieved by using demethylating agent such as 5-aza-2′-deoxycytidine (5-azadC). Therefore, identification of DNA methylation patterns in cancer may be helpful in predicting prognosis and response to chemotherapeutic agents. In addition, to the best of our knowledge there have been very limited studies which investigate global DNA methylation profile in recurrent CRC. Moreover, at the time this research was conducted there is no study which investigate the global methylation changes in *in vitro* CRC model upon treatment with combination of 5-FU with 5-azadC. Thus, the aim of the study was to investigate the methylation phenotype in CRC on the global scale using the high density Infinium DNA Methylation 450K DNA assay in order to identify predictive markers for chemotherapy response. The second aim was to investigate the magnitude of 5-azadC in enhancing 5-FU chemosensitivity and to determine the global methylation changes in cells treated with 5-FU, 5-azadC or combination of both agents.

## Materials and Methods

### Clinical Samples, Cell Lines, and Culture Condition

This study was carried out in accordance with the recommendations of Universiti Kebangsaan Malaysia Research Ethics Committee (Reference number: UKM 1.5.3.5/244/UMBI-001-2014) with written informed consent from all subjects. All subjects gave written informed consent in accordance with the Declaration of Helsinki. The protocol was approved by the Universiti Kebangsaan Malaysia Research Ethics Committee. Forty eight consented CRC patients from Hospital Universiti Kebangsaan Malaysia (HUKM), Malaysia were included in this study; 43 samples were non-recurrent and five were recurrent cases. All patients were Malaysian citizen diagnostically confirmed with primary or recurrent CRC without other cancer history. The tumor samples were from patients who underwent surgery for CRC and have not yet started chemotherapy while those associated with recurrent disease were obtained after the chemotherapy. Formalin fixed, paraffin embedded (FFPE) tissues were collected if frozen tissues were not available.

HT29 and SW48 cell lines were obtained from American Type Culture Collection (ATCC) and maintained in McCoy’s 5A (Gibco) and DMEM (Gibco), respectively at 37°C and 5% CO_2_ in incubator (Galaxy 170R, Eppendorf). Both culture media were supplemented with 10% fetal bovine serum (FBS) (Gibco).

### Tissue Processing and DNA Extraction

The surgically resected tissues were immediately frozen in liquid nitrogen and stored at -80°C before further analysis. All tissues were sectioned and stained with Hematoxylin and Eosin (H&E). Only tissues that contain more than 80% of tumor cells were used. DNA from fresh frozen tissues and cell lines were extracted using QIAamp DNA mini kit while QIAamp DNA FFPE tissue kit (both from Qiagen) was used for FFPE specimens according to the manufacturer’s instructions. The quantification and purity of total DNA for each sample were determined by using Nanodrop ND-2000 spectrophotometer (NanoDrop Technologies, Inc.). Only samples with purity from 1.8 to 2.05 were selected for the microarray study.

### Drugs Treatment and Cell Viability Assay

The cells were seeded in 96-well plates at 1 × 10^4^ cells/well and incubated with chemotherapeutic drug 5FU, demethylating agent 5-azadC (both from Sigma) or combination of both for 72 h at different concentration (ranges from 10 to 100 μM) (Flis et al., 2014) and the culture media was replaced every 24 h with fresh media. All agents were dissolved in dimethyl sulfoxide (DMSO) (Nacalai Tesque) and then diluted in the culture media for experiment. The untreated cells served as a control and were treated simultaneously with DMSO (Nacalai Tesque) with final concentration of 0.2%.

### DNA Methylation Profiling and Data Analysis

In all cases, 500 ng of DNA was chemically modified to convert all unmethylated cytosine to uracil by the EZ DNA methylation – Gold kit (Zymo Research, Inc.) according to the manufacturer’s protocol. The Infinium DNA Methylation 450K assay was performed according to the manufacturer’s specifications (Illumina, Inc.). The Illumina Infinium DNA methylation 450K assay examines the DNA methylation status of 485577 CpG dinucleotides distributed over the whole genome.

The raw idat files obtained from methylation microarray were analyzed using the CHAMP Bioconductor packages (Morris et al., 2014; Butcher and Beck, 2015). Filters were applied to all datasets where CpG sites that had detection *P*-values of greater than 0.01 in one or more samples were excluded from further analysis. The raw intensities were SWAN-normalized to reduce the technical biases inherent in the probe design before statistical analysis (Maksimovic et al., 2012). Once normalization has been performed, β-values were extracted. Statistical analysis was performed on the β-value. Differentially methylated CpG sites were determined using *t* statistics from the *limma* Bioconductor package (Morris and Beck, 2015). We further used the filtering characteristic of *P*-value at *P* < 0.05 to identify significant differentially methylated genes.

### Validation of Methylation Microarray Using MS–qPCR

A total of five significant genes differentially methylated in recurrent versus non-recurrent samples CCNE1 (EPSH107315-1A), DDX43 (EPSH112573-1A), CHL1 (EPSH110000-1A), PON3 (EPSH113282-1A), and CCNDBP1 (EPSH104489-1A) were selected from the DNA methylation profile for validation using EpiTect Methyl II PCR assays (SABiosciences) following the manufacturer’s protocol. Methylation-sensitive (EPHS115450-1A) and methylation-dependent (EPHS115451-1A) digest control assays were performed on 34 fresh tissue samples (31 non-recurrent and 3 recurrent tumors). Digested DNA was used as template for qPCR assay using RT^2^ SYBR Green ROX qPCR Mastermixes (SABiosciences, #330520) under standard amplification conditions on Applied Biosystem 7500 Fast Real-Time PCR system (Applied Biosystems, Inc.). Data generated by MS-qPCR were further analyzed as recommended by the manufacturer^1^. Meanwhile, two FFPE samples of recurrent tumor were validated using methylated and unmethylated primers designed from AIT Biotech, Singapore. The bisulfite conversion was performed using EpiTect Bisulfite Kit (SABiosciences) according to the manufacturer’s instruction. MS-qPCR was carried out using Methylamp ^TM^ MS-qPCR Kit (Epigentek Group, Inc.) according to the manufacturer’s protocol on Applied Biosystem 7500 Fast Real-Time PCR system (Applied Biosystems, Inc.).

### Statistical Analysis

For cell viability analysis, all statistical analyses were performed using GraphPad Prism 6.0 statistical software. Experimental data was presented as mean ± standard deviation (S.D). Cell viability was analyzed by two-way ANOVA test. *P* < 0.05 was assumed statistically significant and marked with asterisks on graphs. All experiments were conducted in triplicates to ensure reproducibility of the results.

## Results

### Distribution of Hyper- and hypomethylated Probes in Recurrent versus Primary CRCs

Clinicopathological characteristic of the 48 tumor samples are tabulated in **Table 1**. Majority of the patients were male and age more than 50 years. For the analysis of microarray methylation data, probe filtering was performed to remove probes that failed to hybridize with a detection *P* > 0.01 in more than one sample and probes that are not represented by a minimum of three beads on the array. This resulted in 426,411 probes for the downstream analysis. We then generated the principal component analysis (PCA) to see the clustering among the samples. The PCA plot showed that recurrent CRCs were clustered distinctly compared to the non-recurrent samples (**Supplementary Figure S1**).

**Table 1:** Clinicopathological characteristics of 48 CRC samples comprised of 43 non-recurrent and 5 recurrent CRCs.

Characteristics	Non-recurrent samples (%)	Recurrent samples (%)
**Gender**		
Female	17 (39.5%)	1 (20%)
Male	26 (60.5%)	4 (80%)
**Age (years)**		
≤50	1 (2.30%)	3 (60%)
>50	42 (97.7%)	2 (40%)
**Ethnicity**		
Malay	19 (44.1%)	3 (60%)
Chinese	22 (51.1%)	2 (40%)
India	1 (2.40%)	
Others	1 (2.40%)	
**Duke’s staging**		
A	3 (7.0%)	–
B	16 (37.2%)	–
C	24 (55.8%)	–
Recurrent		5 (100%)

**FIGURE 1:**
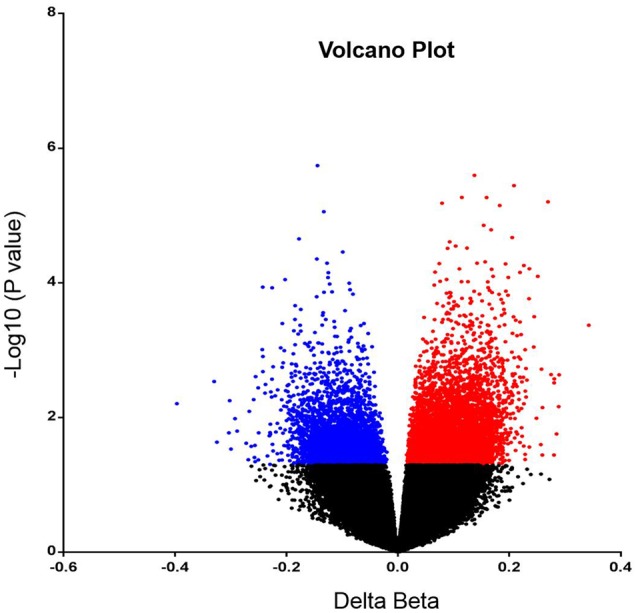
**Volcano plot representing differentially methylated probes.** The significantly hypermethylated probes are shown in red while hypomethylated probes are shown in blue (*P* < 0.05).

**FIGURE 2:**
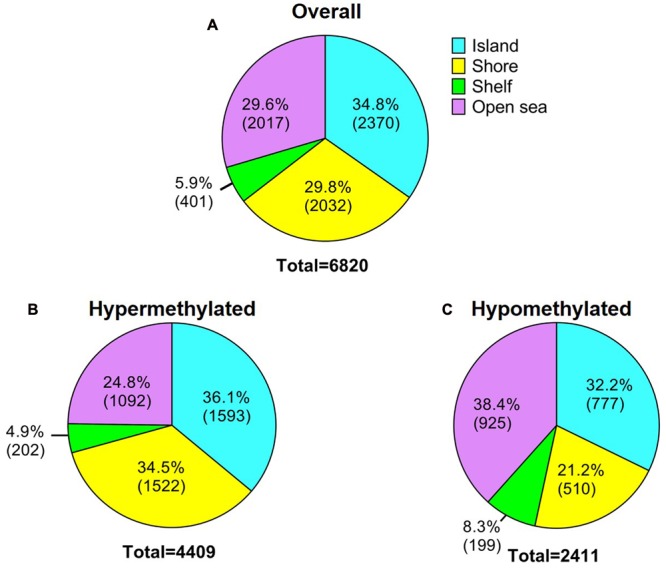
**Description of the 450K DNA methylation array. (A)** General overview of significantly differentially methylated probes in relation to CpG content and neighborhood context. **(B)** Hypermethylated probes and **(C)** Hypomethylated probes according to CpG content and neighborhood context.

In order to analyze DNA methylation differences between recurrent and non-recurrent tumors, we examined the average β-values between both groups. A total of 6820 probes were significantly differentially methylated between recurrent and non-recurrent tumor. Each differentially methylated genes were visualized in the volcano plot (**Figure 1**). Of these 6820 differentially methylated CpGs, 4409 were hypermethylated and 2411 were hypomethylated (**Figure 2A**). From the 4409 differentially hypermethylated probes, 1593 probes were located in the island region, 1522 probes were located in the shore region, 202 probes were in the shelve region and the remaining 1092 probes were in the open sea (**Figure 2B**). In contrast to hypermethylation, most of the hypomethylated CpGs were located in the open sea area of genome followed by island, shores and shelves with 925, 777, 510, and 199 probes, respectively (**Figure 2C**).

**FIGURE 3:**
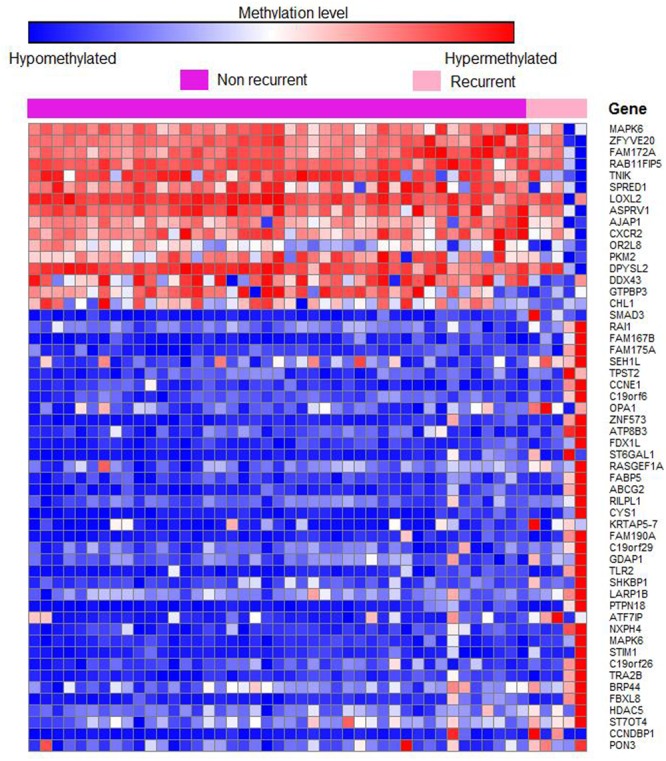
**Heatmap of 50 differentially methylated genes in recurrent versus non-recurrent cases.** Samples were clustered based on 4787 significant differentially methylated genes at *P* < 0.05. The row represents individual genes and the column represents individual samples. The pink color indicates recurrent cases and purple color indicates non-recurrent cases. The color in each small boxes represents the methylation level of the genes. The red boxes indicate genes that are hypermethylated while blue boxes represent genes that are hypomethylated.

**FIGURE 4:**
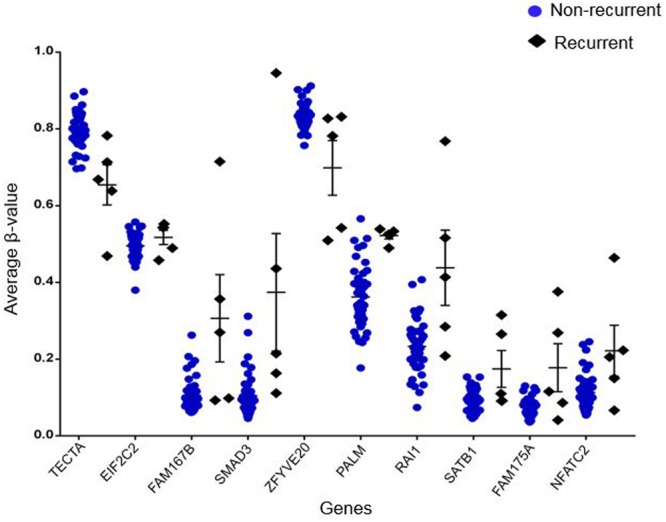
**Top 10 of significantly differentially methylated genes.** The blue circles represent average β-value of respective genes in non-recurrent samples while black diamonds represent average β-value of respective genes in recurrent samples.

Out of 6820 differentially methylated probes, those hypermethylated in recurrent tumor compared to non-recurrent tumor were associated with 3112 genes while 1675 genes were hypomethylated in recurrent samples. Heatmap of the significantly differentially methylated genes in recurrent versus primary CRC is shown in **Figure 3**. Our genome-wide methylation study in CRC showed that recurrent CRCs exhibited increased methylation level compared to non-recurrent CRCs. Fifty eight and 47 of the significantly hypermethylated and hypomethylated genes have an absolute recurrent/non-recurrent methylation difference of ≥20% (Supplementary Table S1-1). The top 10 significantly differentially methylated genes are TECTA, EIF2C2, FAM167B, SMAD3, ZFYVE20, PAM, RAI1, SATB1, FAM175A, and NFATC2 (average Δβ-value between recurrent and non-recurrent group of -0.144, 0.137, 0.208, 0.269, -0.132, 0.167, 0.205, 0.093, 0.104, and 0.124, respectively) (**Figure 4**).

### Pathways Enrichment analyses of Differentially Methylated Genes

Next, the list of hypermethylated and hypomethylated genes were subjected to pathway enrichment analysis using the DAVID (Huang et al., 2003, 2009). We found five significant enriched pathways of hypermethylated genes that are potentially involved in the recurrent CRCs, namely, pathways in cancer, MAPK signaling pathway, focal adhesion, calcium signaling pathway and Wnt signaling pathway (**Table 2A**). The MAPK signaling pathway which is known to be associated with CRC, showed hypermethylation of 56 genes that were mostly regulating the apoptosis process.

**Table 2:** Five top pathways regulated by the hypermethylated genes in recurrent CRCs compared to non-recurrent.

Pathway	No. of genes	*P*-value	Enrichment score
**(A)**

Pathway in cancer	68	0.013	1.304
MAPK signaling pathway	56	0.020	1.319
Focal adhesion	44	0.021	1.377
Calcium signaling pathway	39	0.025	1.394
Wnt signaling pathway	37	0.006	1.542

**(B)**

Pathway in cancer	40	0.036	1.362
Neuroactive ligand-receptor interaction	37	0.003	1.615
Endocytosis	31	7.58E-04	1.882
Regulation of actin cytoskeleton	31	0.008	1.611
Focal adhesion	29	0.011	1.612

**FIGURE 5:**
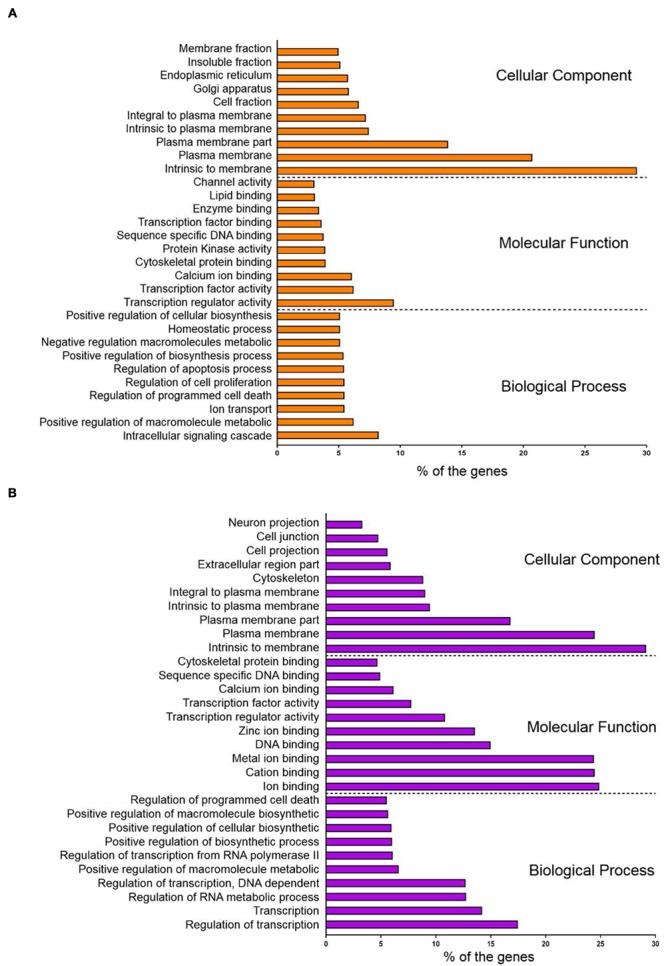
**Gene ontology (GO) enrichment analysis of (A)** hypermethylated genes and **(B)** hypomethylated genes with. The genes were clustered according to the cellular component, molecular function and biological process.

Conversely, the hypomethylated genes in recurrent CRCs were associated with pathways in cancer, neuroactive ligand-receptor interaction, endocytosis, regulation of actin cytoskeleton and focal adhesion (**Table 2B**). For the pathways in cancer, the genes specifically involved are in the PI3K-AKT signaling pathway and proliferation process. The genes involved in this signaling pathway and proliferation process include PI3K, PPFP, RXR, EGFR, CCNA1, and E2F. We also performed gene ontology (GO) enrichment analysis to classify the hyper- and hypomethylated genes into the categories of cellular component, biological process and molecular function. In the cellular component category, 25–30% of the hyper- and hypomethylated genes are shown to be intrinsic to membrane (**Figure 5**). For the molecular function and biological process, the hypermethylated genes were enriched for transcription regulator activity and intracellular signaling cascade (**Figure 5A**), while the hypomethylated genes were enriched for ion binding and regulation of transcription (**Figure 5B**).

### Validation of the HumanMethylation450 Beadchip Array

**FIGURE 6:**
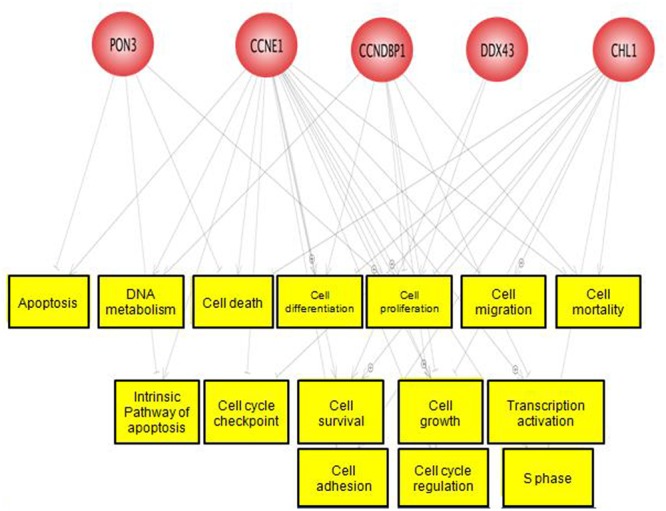
**Predicted function of the five selected genes.** These genes were mostly involved in cell cycle, cell growth, and cell differentiation.

A total of three differentially hypermethylated genes (CCNE1, PON3, and CCNDBP1) and two differentially hypomethylated genes (DDX43 and CHL1) were selected for the validation based on their β-value and association with CRC as well as other cancers. We explored the function of these genes using the Pathway Studio software. These genes were mostly involved in cell cycle, cell proliferation and cell growth as shown in **Figure 6**. The methylation pattern of validation using EpiTect Methyl II PCR assay were in concordance with methylation profiling (**Supplementary Figure S2**).

### Growth Studies and Effects of Combination Treatments of 5-FU with 5-azadC

**FIGURE 7:**
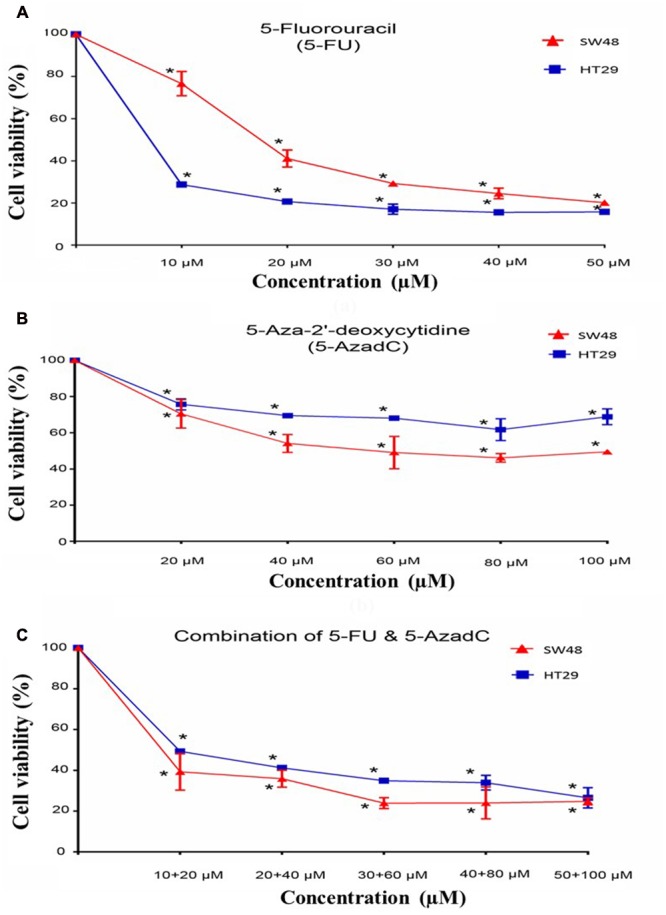
**Growth studies and effects of combination treatments of 5-FU with 5-azadC.** Treatment of **(A)** 5-Fluorouracil (5-FU), **(B)** 5-Aza-2′-deoxycytidine (5-AzadC), **(C)** combination of 5-FU and 5-azadC on SW48 and HT29 for 72 h. Data is presented as mean ± SD, *n* = 9. ^∗^*P* < 0.05 compared to untreated cells.

Cell viability assay was performed on HT29 and SW48 after treatment with 5-FU, 5-AzadC or combination of both drugs. Firstly, the effect of 5-FU alone on HT29 and SW48 cell viability was examined. Both cells were treated with concentration ranges from 10 to 50 μM. The results of cell viability assays indicated that HT29 showed more inhibition of cells as the concentration of 5-FU increased compared to SW48 (**Figure 7A**). The decrease of HT29 cell viability is more than 50% even at lowest concentration (10 μM). For SW48, the cell viability decreased only 25% at the 10 μM and only reached 50% inhibition at higher dose (20 μM 5-FU). We also observed the effect of 5-AzadC on both cells at concentration ranges from 20 to 100 μM for 72 h (**Figure 7B**). Inhibition of cell viability by 5-AzadC was more prominent in SW48 compared to HT29 (30–50% versus 25–30%). Nonetheless, all the cells treated with either drugs showed significant reduction in cell viability compared to the untreated cells (*P* < 0.05).

Apart from that, we also examined the effect of 5-FU in combination with 5-AzadC on CRC cell survival at various concentrations. The effect of combining both drugs at lowest concentration is more promising in SW48 cells (60% inhibition) compared to the treatment with 5-FU (25% inhibition) or 5-AzadC alone (30% inhibition) (**Figure 7C**). This showed that 5-azadC enhanced the inhibitory effects of 5-FU in SW48 even at the lowest concentration tested (10 μM). On the contrary, combinatorial drugs treatment on HT29 cells showed lower reduction in cell viability (50% inhibition) compared to 5-FU alone (70% inhibition).

### Global Methylation Response to 5-FU and 5-azadC

Following the treatment of cells with all drugs over 72 h, methylation profiling was performed on both cells in order to identify methylation pattern cause by the chemotherapeutic and/or demethylating agent. Methylation profiling of combined 5-FU and 5-AzadC treatment was performed on SW48 and HT29 to observe whether combination treatment would reverse the process of methylation or remains unchanged. Moreover, we also performed methylation profiling on cells that treated with 5-FU or 5-AzadC only in order to compare the methylation level of combined treatment. The cells treated with 20 μM of 5-FU and 40 μM of 5-AzadC were chosen based on the concentration at which cell viability was reduced by 50%.

Treatment with 5-FU in HT29 caused 70% reduction in cell viability with significant hypermethylation of only five genes and hypomethylation of four genes (Supplementary Tables S1-1 and S1-2). On the other hand, 5-azadC not only caused significant hypomethylation of 329 genes but also induced hypermethylation of 172 genes (Supplementary Table S1-3). Despite higher number of aberrantly methylated genes, the impact on cell viability is lesser compared to 5-FU (30%). Combination of both 5-FU and 5-azadC resulted in 114 hypermethylated and 68 hypomethylated genes (Supplementary Tables S1-1–S1-4) with 55% reduction in cell viability. Since 5-FU alone was able to reduce cancer cell viability in HT29 even at the lowest concentration tested (10 μM), chemosensitization using 5-azadC is deemed unnecessary.

**FIGURE 8:**
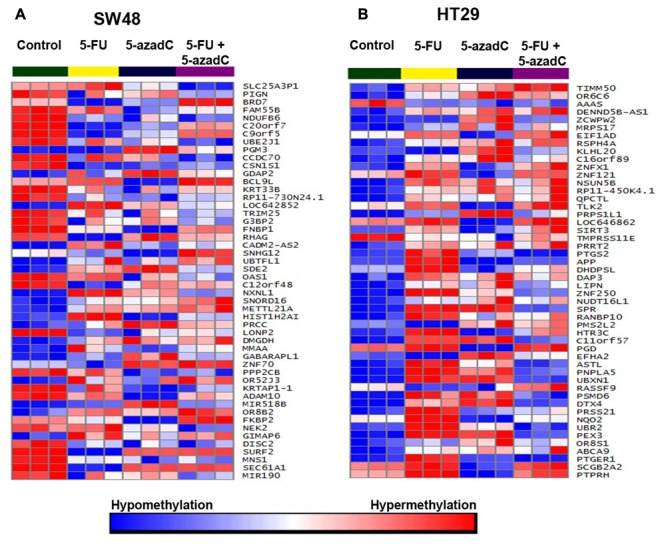
**Methylation profiling of SW48 and HT29 cell lines.** Methylation profiling was carried out following incubation with 5-FU, 5-aza-dC and combination of both drugs. Untreated cells were included as control. **(A)** Heatmap of the 50 top significantly differentially methylated genes in SW48. Red represent hypermethylated genes while blue represent hypomethylated genes. **(B)** Heatmap of the top 50 significantly differentially methylated genes in HT29.

At 20 μM, 5-FU treatment in SW48 caused 60% reduction in cell viability with hypermethylation of 313 genes and hypomethylation of 49 genes (Supplementary Tables S1-1–S1-5). On the other hand, 5-azadC caused significant hypomethylation of 520 genes but also induced hypermethylation of 83 genes (Supplementary Tables S1-1–S1-6). Similarly with HT29, although the number of aberrantly methylated genes was higher, the impact on cell viability is lesser compared to 5-FU (55%). Combination of both 5-FU and 5-azadC resulted in even greater number of differentially methylated genes; 762 genes were hypermethylated and 589 were hypomethylated (Supplementary Tables S1-1–S1-7) with profound reduction in cell viability compared to 5-FU alone. Collectively, these results showed that 5-azadC has successfully sensitized SW48 to 5-FU even at lowest concentration tested (10 μM). Subsequent pathway enrichment analysis revealed MAPK signaling pathway as one of the significantly enriched pathway in this combinatorial drug treatment (**Table 3**). **Figure 8** illustrate the global methylation changes in various treatment groups compared to untreated control cells.

**Table 3:** Five top pathways regulated by the hypomethylated genes in SW48 treated with combination drugs of 5-FU and 5-AzadC.

Pathway	No. of genes	*P*-value	Enrichment score
Pathway in cancer	203	0.010	1.187
MAPK signaling pathway	173	0.080	1.219
Focal adhesion	129	0.050	1.207
Endocytosis	117	0.084	1.196
Axon guidance	91	0.013	1.327

## Discussion

Many studies have shown that DNA methylation is an important underlying mechanism for drug resistance which subsequently posed the increased risk of recurrence. Despite a growing consensus that methylation is a modulator of cancer, the understanding of DNA methylation patterns of recurrent CRC remains limited. To the best of our knowledge, there is no publication addressing the genome-wide DNA methylation analyses of recurrent CRC using the Illumina Infinium HumanMethylation450 Beadchips. In our study, we identified 4787 significantly differentially methylated genes (*P* < 0.05) on methylation profiling of 43 non-recurrent and five recurrent samples; 3112 genes were hyper- while 1675 genes were hypomethylated. Our results showed that recurrent CRC exhibit increased methylation levels compared to non-recurrent group. Similar patterns have been observed in chordomas where higher methylation level were observed in recurrent chordomas compared to non-recurrent (Alholle et al., 2015).

Most of the hypermethylated genes were commonly observed in the CpG islands which are associated with promoter regions. Our results are supported by a review from Jin et al. (2011) where most studies showed hypermethylation occurring at CpG-rich promoter regions. By contrast, hypomethylation often occurs in the open sea area of the genome. McCabe et al. (2009) reported that hypomethylation were observed in the intergenic region as well as the gene body (Zheng, 2015) in which these two regions are located in the open sea area (Ching et al., 2015). Pathway enrichment analysis of hyper- and hypomethylated genes revealed that most of the hypermethylated genes of recurrent CRCs were involved in the MAPK signaling pathway which specifically targets the apoptosis process. Cells which are able to escape from the apoptosis process are associated with chemotherapy resistance thus will increase the risk of recurrent cancer (Kasibhatla and Tseng, 2003). In a few studies, TGFβ, CASP3, DAXX, and p38 were found to be hypermethylated and these genes are known to regulate the apoptosis process (Wagner and Nebreda, 2009; Lee et al., 2012; McIlwain et al., 2013; Pan et al., 2013). This is supported by Perry et al. (2006) where most of the hypermethylation in cancer occurred in the genes that regulate the apoptosis process and other cellular functions. On the contrary, the hypomethylated genes of recurrent CRCs are involved in the pathways in cancer particularly in regulating the cell proliferation process and PI3K-AKT signaling pathway. Among the genes involved in the proliferation process and signaling pathway are PPFP, RXR, EGFR, PI3K, CCNA1, and E2F (Balasubramanian et al., 2004; Yamazaki et al., 2005; Rolland, 2006; Yuan and Cantley, 2008; Webster et al., 2009; Reddi et al., 2010). Hypomethylation could increase the expression of these genes (Ehrlich, 2009) leading to the activation of PI3K-AKT signaling pathway and proliferation process. The activation of this pathway may increase the cell survival, hence resulting in the increased risk of recurrence.

Three hypermethylated genes (CCNE1, CCNDBP1, and PON3) and two hypomethylated genes (DDX43 and CHL1) were selected for validation. These genes are involved in cell cycle, cell proliferation and cell growth. CCNE1 belongs to the highly conserved cyclin family and is an important component in the cell cycle regulation. CCNE1 forms a complex with CDK2 and is involved in the transition of G1 to S phase as well as in the DNA replication (Hwang and Clurman, 2005). Increased expression of this gene can cause chromosomal instability and lead to the formation of cancer such as breast cancer, leukemia, non-small lung cancer, and others (Moroy and Geisen, 2004). However, in this study we found that CCNE1 is hypermethylated and its expression was downregulated in recurrent CRCs compared to non-recurrent. This finding is in concordance with the study by Kleivi et al. (2007) where CCNE1 expression was lower in the carcinomatosis group compared to primary cancer. The decreased expression of this gene from primary CRC is associated with poor prognosis, advanced stage and metastasis (Li et al., 2001). Apart from that, a study from Nakayama et al. (2010) showed that increased expression of CCNE1 in primary ovarian cancer was associated with chemotherapy resistant and increased risk of recurrence. Therefore, we believed that hypomethylation of this gene in non-recurrent CRCs is associated with chemotherapy resistant which subsequently contribute to recurrent CRCs. However, further investigation is warranted to confirm this hypothesis.

Cyclin D type binding protein 1 (CCNDBP1), also known as GC1P, is a tumor suppressor gene that is downregulated in some cancers such as breast and prostate (Mostoslavsky et al., 2006; Michishita et al., 2008). In colon cancer, the expression of this gene is lower in tumor tissues compared to normal (Ma et al., 2007). Our study showed that *CCNDBP1* is hypermethylated in recurrent CRCs compared to non-recurrent, which could possibly explain the lower expression of this gene. This is the first report of hypermethylation of *CCNDBP1* in recurrent CRCs. Paraoxonase 3 (PON3) belongs to the paraoxonase families (PON1, PON2, PON3) that helps in preventing oxidative stress and anti-inflammatory. This gene is also involved in other diseases including cancer (Devarajan et al., 2014). PON3 gene has high expression in cancer tissues of the lung, liver and colon (Schweikert et al., 2012). A study by Schweikert et al. (2012) showed that the expression level of PON3 is different in various stages of cancer. Stage I exhibit decreased expression of PON3 while stage II and III display increased expression of this gene (Schweikert et al., 2012). In our study, we identified hypermethylation of PON3. This is in line with the study by Alholle et al. (2015), where PON3 was hypermethylated in recurrent chordomas compared to the primary tumor. DDX43, also known as helicase antigen (HAGE), is responsible for cell proliferation and has a high expression level in cancer compared to normal (Abdel-Fatah et al., 2014). Our microarray profiling shows that DDX43 is hypomethylated in recurrent CRCs. Hypomethylation of DDX43 induces the expression of the gene and is strongly correlated with advanced disease and poor prognosis in leukemia (Lin et al., 2014). DDX43 expression is increased in 50% of acute myeloid leukemia cases (Adams et al., 2002) and high expression will activate the Ras signaling pathway thus promoting cell proliferation (Ambrosini et al., 2014). The dysregulation of this process may cause the progression of cancer such as metastasis (Linley et al., 2012).

Close Homolog of L1 (CHL1) is important for brain development and neuron activities (Manderson et al., 2009). It also plays a role in cancer progression (Qin et al., 2008) and contributes to the metastasis process (Siesser and Maness, 2009). In recurrent CRCs, we identified hypomethylation of this gene compared to non-recurrent CRCs. Hypomethylation of *CHL1* was also observed in metastatic oral squamous cell carcinoma (OSCC) compared to non-metastatic tumor (Huang et al., 2013). A previous reported the low expression of CHL1 in majority of primary cancers and its expression is increased in the metastatic or invasive tumor (Senchenko et al., 2011).

The potential of demethylating agent such as 5-azadC in sensitizing resistant cancer cells to chemotherapeutic agent in CRC *in vitro* model has already been studied by several groups (Fang et al., 2004; Mossman et al., 2010; Khamas et al., 2012; Flis et al., 2014). Nevertheless, the global methylation changes following the treatment is not well-characterized and the aforementioned studies pre-selected certain genes for further investigation. The action of 5-azadC is not gene-specific and vary across different cell lines thus it is important to know which genes or pathway will be affected by this agent. Furthermore, the chemotherapeutic agent used by previous studies was Trichostatin A (TSA) (Fang et al., 2004; Mossman et al., 2010) which is not commonly practiced by our oncologist. To be more relevant with our population, we investigated the effect of 5-FU chemosensitization by 5-azadC in hypermethylated and hypomethylated cell lines and profiled the global methylation changes caused by the agents whether singular or in combination. We observed that the reduction in cancer cell viability is significantly greater in SW48 compared to HT29 when treated with both 5-FU and 5-azadC. This is in concordance with other study which showed that there was an increase in inhibition of CRC cells growth after combinatorial treatment with both agents (Flis et al., 2014).

SW48 cells treated with combined drugs showed decrease in methylation level, particularly in genes that involved in MAPK signaling pathway which specifically target p53 signaling pathway. P53 is a tumor suppressor gene that helps in regulates apoptosis process and cell cycles (Nieto et al., 2004). DNA damage induced by chemotherapy have been shown to activate p53 signaling pathway which can trigger the apoptosis process. Our study revealed demethylation of genes involve in p53 signaling pathway in SW48 which then could possibly induce the activation of these genes. As these genes become activated, the presence of 5-FU will cause DNA damage and therefore p53 signaling pathway could be re-activated and lead to apoptosis process. Hypomethylation of genes involved in Fas mediated apoptosis pathway which were FAS, DAXX, ASK1, and MAX was also observed in SW48 treated with combined drugs. DAXX gene will bind to FAS death domain, and overexpression of DAXX will initiates Fas-induced apoptosis through the activation of ASK1, MKK6, p38, and MAX genes (Yang et al., 1997).

This study has several obvious limitations. First, the number of recurrent CRC cases used in this study is small thus we compensated with increasing the sample size of the non-recurrent CRCs and performed genome-wide profiling instead of targeting specific genes. Secondly, we did not manage to obtain a matched primary and recurrent cancer tissues from the same patient. It would be ideal to perform the comparison using the cancer tissues from the same individual.

## Conclusion

To the best of our knowledge, our study is the first to address the genome wide methylation changes in recurrent CRCs and non-recurrent CRCs. The increased in global methylation levels were observed in the recurrent cases. Our study revealed several potentially biologically important genes – CCNE1, CCNDBP1, PON3, CHL1, and DDX43 that may represent new potential therapeutic targets for patients with chemoresistant phenotype. Our *in vitro* findings suggest that 5-azadC-mediated restoration of 5-FU sensitivity is mediated at least in part by MAPK signaling pathway. Further investigation is needed in order to completely understand the involvement of these genes in the cancer recurrence.

## Ethics Statement

This study was carried out in accordance with the recommendations of Universiti Kebangsaan Malaysia Research Ethics Committee (Reference number: UKM 1.5.3.5/244/UMBI-001-2014) with written informed consent from all subjects. All subjects gave written informed consent in accordance with the Declaration of Helsinki. The protocol was approved by the Universiti Kebangsaan Malaysia Research Ethics Committee.

## Author Contributions

RB performed the experiment as described in the manuscript, statistical analysis, interpretation of the data and drafted the manuscript. N-SAM performed interpretation of the data and gave critical insight to the manuscript. SO assisted in the analysis of the methylation profiling data. IS is a colorectal surgeons which assisted in specimen retrieval. IR is a pathologist which evaluated the histopathology of the specimens. NM and RJ are mentors, assisted in coordination of the study and gave critical evaluation of manuscript. All authors read and approved the final manuscript.

## Conflict of Interest Statement

The authors declare that the research was conducted in the absence of any commercial or financial relationships that could be construed as a potential conflict of interest.

## Footnotes

1 http://www.sabiosciences.com/dna_methylation_data_analysis.php/

## Supplementary Material

FIGURE S1 **Principle Component Analysis (PCA) of the raw methylation data. Non-recurrent tumors (indicated by yellow color) were clustered distinctly from recurrent group (indicated by red color)**.

FIGURE S2 **Validation of methylation array profiling. The result from the validation experiment is in concordance with methylation profiling.** The gray bars indicate the percentage of methylation of respective genes in the recurrent samples while blue bars represent percentage of methylation in the non-recurrent samples.
