# Supplementary figures and images for: Identification of Predictive DNA Methylation Biomarkers for Chemotherapy Response in Colorectal Cancer
Source: Front Pharmacol. 2017 Feb 13;8:47. doi: 10.3389/fphar.2017.00047 (PMC5303736; doi:10.3389/fphar.2017.00047)

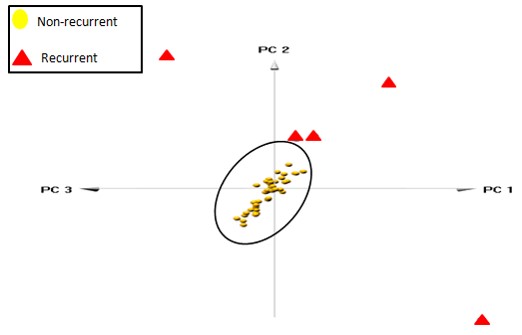

Supplement: FIGURE S1 — Principle Component Analysis (PCA) of the raw methylation data. Non-recurrent tumors (indicated by yellow color) were clustered distinctly from recurrent group (indicated by red color). [file Image_1.JPEG]

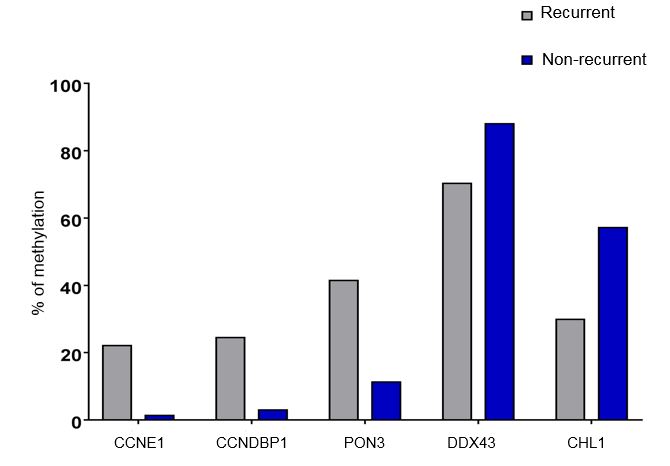

Supplement: FIGURE S2 — Validation of methylation array profiling. The result from the validation experiment is in concordance with methylation profiling. The gray bars indicate the percentage of methylation of respective genes in the recurrent samples while blue bars represent percentage of methylation in the non-recurrent samples. [file Image_2.JPEG]
